# Fully Integrated Passive UHF RFID Tag for Hash-Based Mutual Authentication Protocol

**DOI:** 10.1155/2015/498610

**Published:** 2015-09-29

**Authors:** Shugo Mikami, Dai Watanabe, Yang Li, Kazuo Sakiyama

**Affiliations:** ^1^Hitachi Ltd., Research & Development Group, Center for Technology Innovation-Systems Engineering, 292 Yoshida-cho, Totsuka-ku, Yokohama, Kanagawa 244-0817, Japan; ^2^Department of Informatics, The University of Electro-Communications, 1-5-1 Chofugaoka, Chofu, Tokyo 182-8585, Japan

## Abstract

Passive radio-frequency identification (RFID) tag has been used in many applications. While the RFID market is expected to grow, concerns about security and privacy of the RFID tag should be overcome for the future use. To overcome these issues, privacy-preserving authentication protocols based on cryptographic algorithms have been designed. However, to the best of our knowledge, evaluation of the whole tag, which includes an antenna, an analog front end, and a digital processing block, that runs authentication protocols has not been studied. In this paper, we present an implementation and evaluation of a fully integrated passive UHF RFID tag that runs a privacy-preserving mutual authentication protocol based on a hash function. We design a single chip including the analog front end and the digital processing block. We select a lightweight hash function supporting 80-bit security strength and a standard hash function supporting 128-bit security strength. We show that when the lightweight hash function is used, the tag completes the protocol with a reader-tag distance of 10 cm. Similarly, when the standard hash function is used, the tag completes the protocol with the distance of 8.5 cm. We discuss the impact of the peak power consumption of the tag on the distance of the tag due to the hash function.

## 1. Introduction

Radio-frequency identification (RFID) has been widely used in various areas. A passive RFID tag sends identification information (ID) to a reader wirelessly. The reader identifies the tag with the ID. Due to the ability to communicate wirelessly, the RFID tags have become widespread within many areas such as supply chain and transportation.

According to the report [1], 5.8 billion tags were sold in the world in 2013, and 6.9 billion tags are sold in 2014. While the RFID market was worth $7.8 billion in the world in 2013, it rises to $8.9 billion in 2014. It is predicted that the RFID market will be worth $27.3 billion in 2024. Most of the growth results from passive UHF (Ultra High Frequency) RFID tags.

On the other hand, there are privacy and security issues on the RFID tags. For example, since the tag sends a fixed ID to a reader whenever the tag receives a query sent from a reader, it allows tracking the owner by tracing the ID and it also allows generating a forged tag.

To overcome these issues, privacy-preserving RFID authentication protocols based on cryptographic algorithms have been developed [2–5]. OSK protocol is one of the protocols based on hash functions [6]. An improved version of OSK protocol, which is called OMHSO protocol, has been developed [7]. The hardware performance of the cryptographic modules or the digital processing blocks that run a part of the authentication process has also been studied.

However, an issue still remains an open problem in the following sense. Implementation and evaluation of the whole tag, which includes an antenna, an analog front end, and a digital processing block, that runs authentication protocols have not been studied. It is very important to evaluate the whole tag since these modules are required to run the whole authentication process and these modules operate in a mutually coupled manner.

In this paper, to tackle this issue, we design and implement a fully integrated passive UHF RFID tag that runs a privacy-preserving mutual authentication protocol based on a hash function. Then, we carry out a full silicon proof of the tag. We show evaluation results of the tag. We design a single chip including the RF front end and the digital processing block. The chip is attached to an antenna. We choose OMHSO protocol to preserve privacy. We select SPONGENT-160 [8], which is a lightweight hash function supporting 80-bit security strength, and Keccak [9], which is a newly selected NIST standard hash function supporting 128-bit security strength, as the hash function used in the protocol. These algorithms are switched and used. To the best of our knowledge, this is the first feasibility evaluation of a fully integrated passive tag that runs a mutual authentication protocol based on a cryptographic hash function.

Our evaluation results show that the tag runs the whole authentication process using SPONGENT-160 under the condition that the distance between the tag and a reader is 10 cm. The protocol is completed within 30 ms. The number of equivalent gates of the cryptographic block is 10 kGE. The average power consumption of the cryptographic block is 260 *μ*W when the hash function module is active. The peak power consumption of the tag is 3.5 mW that is consumed by EEPROM access.

When Keccak is used as the hash function, the tag runs the whole authentication process under the condition that the distance is 8.5 cm. The protocol is completed within 20 ms. The number of equivalent gates of the cryptographic block is 39.4 kGE. The average power consumption of the cryptographic block is 536 *μ*W when the hash function module is active. The peak power consumption of the tag is 14.3 mW that is consumed by hash calculation.

While it has been widely believed that the lightweight cryptographic algorithms are suitable for RFID tags, performance advantages of the tag using these algorithms against the standard cryptographic algorithms have not been cleared. Our results show that the maximum read distance of the tag using the lightweight cryptographic algorithm becomes about 1.5 cm longer than that of the tag using the standard cryptographic algorithm. When the lightweight hash function is used, the power consumption of the EEPROM is a bottleneck. Therefore, the maximum read distance will be longer when a low-power memory will be used on the tag. On the other hand when the standard hash function is used, the power consumption of the hash function module is a bottleneck. Therefore, the maximum read distance will not be longer even if a low-power memory will be used.

The rest of this paper is organized as follows.  Section 2 reviews the previous works about the RFID system.  Section 3 shows a specification of a fully integrated passive tag.  Section 4 reports the evaluation results of the tag.  Section 5 summarizes the paper.

## 2. Previous Works

To overcome security and privacy issues, privacy-preserving RFID authentication protocols based on cryptographic algorithms have been developed [11]. OSK protocol is a privacy-preserving authentication protocol based on hash functions and it is proposed by Ohkubo et al. in 2002 [6]. The tag executes as follows. A secret key, which is stored in the tag's nonvolatile memory, is taken as the input to one hash function *H*_2_. The tag returns the output of *H*_2_. At the end, the secret key is taken as the input to the other hash function *H*_1_, and the key is updated with the output of *H*_1_.

These protocols help enhance the privacy and the security. On the other hand, from a user's point of view, the communication distance between a tag and a reader is an important issue. The communication distance has negative correlation with the power consumption of the tag by Friis' formula [12]. The available power on the tag is very limited since the tag should generate power needed for modules from only the RF signal transmitted by a reader and the transmission power of the reader is limited by the law. For example, the upper bound of the transmission power is 250 mW in Japan and 500 mW in EU. Therefore, from a designer's point of view, it is important to design a low-power tag.

Since cryptographic components, which form a building block of the protocols, increase power consumption and area requirements of the tag, hardware performance of the cryptographic modules or the digital processing block that runs a part of the authentication process based on cryptographic algorithms has been studied. ASIC (Application Specific Integrated Circuit) implementation results of lightweight cryptographic algorithms have been discussed [13, 14]. In [15], FPGA (Field Programmable Gate Array) implementation results of the digital processing block that executes a hash-based challenge-response protocol have been studied. In [16], implementation results of OSK protocol on an IC card have been studied. In [17], ASIC implementation results of the digital processing block including block cipher AES [18] and stream cipher Grain [19, 20] have been studied. In [21], ASIC implementation results of CRYPTOGPS including lightweight block cipher PRESENT [22] have been studied.

In industry, some semiconductor manufacturers produce RFID tags with cryptographic components. ORIDAO produces a secured EPC Gen2 chip including 192-bit hash function [23]. NXP semiconductors sell MIFARE DESFire EV1 using block cipher 3DES [24]. (MIFARE and DESFire are registered trademarks of NXP Semiconductors.).

In [25], Martin et al. have focused on two lightweight mutual authentication protocols. These protocols are based on a PRNG (pseudorandom number generator) and simple functions such as rotation operation. They have designed a digital circuit including the PRNG, registers, and control logic. They have synthesized the circuit and evaluated hardware performance of the circuit in the sense of area requirements, power, and throughput. In [26], Liu et al. have proposed a lightweight mutual authentication protocol. The protocol requires a random number generator and a LFSR (linear feedback shift register). They have designed a digital circuit including the LFSR and control logic. They have synthesized the circuit and evaluated hardware performance of the circuit in the sense of area requirements and power by performing a postlayout simulation.

They have implemented the digital circuit and have evaluated hardware performance of the digital circuit in the sense of area requirements and power consumption by performing a simulation. However, they have not carried out a silicon proof of the circuit and have not evaluated hardware performance of a tag including an antenna that is attached to the circuit to communicate over the air with a reader.

The authentication protocol implemented in the tag requires additional components such as an analog front end, RF module, EEPROM, and an antenna. Therefore, we design a completely assembled tag including not only a digital circuit but also the above-mentioned components. We have carried out a full silicon proof of the tag. The silicon proof of the tag enables us to evaluate hardware performance of the tag in the sense of the maximum read distance and the operation time. These metrics have a strong impact on the usability of the tag.

Reference [10] has introduced an implementation result of a passive UHF RFID tag chip that runs OMHSO protocol. The feasibility of the chip has been evaluated under the condition that the RF signal is generated by an RF signal generator. As the cryptographic component, SPONGENT-160 has been implemented.

## 3. Specification of a Fully Integrated Passive Tag

### 3.1. Overview

Unlike [10], we implement a fully integrated passive UHF RFID tag that runs OMHSO protocol in this paper. We design a single chip including the RF front end and the digital processing block. If these two kinds of units are implemented separately on the tag, the power consumption of the tag is predicted to be large since an electric current is required to make flow between the chips. To reduce the electric current between the analog part and the digital part, we have decided to integrate the analog front end and the digital processing block in a single chip. Our tag has the ability to communicate with a reader on the market without extra equipment. As the cryptographic component, we implement not only SPONGENT-160 but also Keccak.

### 3.2. Authentication Protocol

While OSK protocol is a privacy-preserving authentication protocol, the protocol is vulnerable to the desynchronization caused by a communication error. The desynchronization problem results in an authentication failure since the secret data stored in the tag and the server does not match. On the other hand, OMHSO protocol, which is a hash function based protocol, has a resistance against the desynchronization by introducing a mutual authentication scheme. Thus, we target OMHSO protocol to enhance the security and the privacy.

#### 3.2.1. OMHSO Protocol

OMHSO protocol is an improved version of OSK protocol and is proposed by Hanatani et al. in 2012 [7]. This protocol is a mutual authentication protocol between the tag and the server. The desynchronization issue caused by a communication error is solved by the mutual authentication scheme using two secret keys that are the current secret key *S*_*i*_ and previous secret key *S*_*i*−1_.

We show the process of the server in Algorithm 1 and the process of the tag in Algorithm 2. *S*_*i*_ and *S*_*i*−1_ are the secret keys of a tag and are shared with a server. *X* and *α* are random numbers generated by the server and the tag, respectively. *H*_0_, *H*_1_, and *H*_2_ are random oracles that are usually substituted for keyed hash functions.

In Algorithms 1 and 2, we show authentication processes of the server and the tag without redundancy. For example, in [7], a flag is used to specify the input data to *H*_1_ to generate *Z*. However, the flag is not necessary to implement the protocol. In addition, we only use secret key and remove ID and counter. This is because the roles of these data are included in the secret key.

### 3.3. Hash Function

In [27], it is recommended that the smallest security strength for general purpose is 80 bits. It is also mentioned that 64-bit security strength supports insufficient protection. To the best of our knowledge, SPONGENT-160 [8], D-QUARK [28], and PHOTON-160/36/36 [29] are the lightweight hash functions supporting 80-bit security strength. We select SPONGENT-160 that achieves low-power consumption [8, 29] in the same manner as [10].

While the cryptographic algorithms supporting 80-bit security strength are suitable for small electric devices because of low-power consumption, these devices will be an important component of the Internet of Things where security plays an important role. NIST shows a guidance to migrate to the use of stronger cryptographic algorithms [30]. In the guidance, migration to 128-bit security strength is required. Thus, we also choose newly selected NIST standard hash function Keccak to support strong security strength.

In summary, as the hash function, we choose lightweight hash function SPONGENT-160 supporting 80-bit security strength and NIST standard hash function Keccak supporting 128-bit security strength.

As described in Section 3.2, three kinds of hash functions and a random number generator are required to run the protocol on the tag. However, separate implementation of these components will result in large area cost of the tag and the tag will consume much power since the power consumption of the circuit is generally proportional to the area requirements of the circuit.

To reduce cost of the tag, we decided to use a minimum cryptographic component that performs the necessary functions. For three hash functions, we decided to use single hash function with different padding manners to realize different hash calculations. For a random number generator, it is quite natural to use a pseudorandom number generator (PRNG). Moreover, as described in [31], a hash function is used as a pseudorandom number generator. Therefore, we only implement single hash function and use it for three hash value calculations and a pseudorandom number generation.

When the hash function is used as the pseudorandom number generator, the seed, which is stored in a nonvolatile memory, is taken as the input to the hash function. The output of the hash function is cut into two parts. One is used as the pseudorandom number. The other is used as the updated seed and is stored in the nonvolatile memory.

### 3.4. Implementation Details

**Figure 1:**
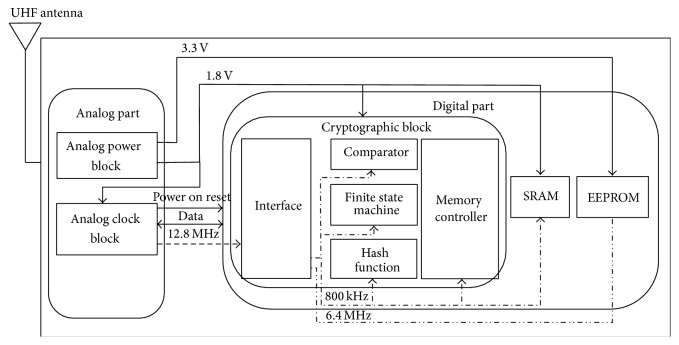
Block diagram of the single chip.

In this section, we describe the functionality and the characteristics of the blocks in the tag chip.  Figure 1 shows the functional blocks of the single chip. We stress here that while we have implemented SPONGENT-160 only in the hash function block in [10], we implement both SPONGENT-160 and Keccak in the hash function block.

The tag chip consists of the analog part and the digital part. The analog part consists of the analog power block and the analog clock block. The analog power block extracts the energy from the RF field and supplies the power for all the rest of the blocks. This block has buffer capacitors. This block generates two supply voltages of 1.8 V and 3.3 V.

The analog clock block generates clock signals needed in the digital processing block. This block has the oscillator and generates 12.8 MHz clock signal that is the source of all the clock signals. This block also performs the demodulation/modulation and power on reset. The input supply voltage of this block is 1.8 V.

The digital part consists of the interface block, the comparator block, the finite state machine block, the hash function block, the memory controller block, the volatile memory, and the nonvolatile memory. The interface block generates the clock signals from 12.8 MHz clock signal that is taken as the input to the block. The circuit in this block allows for lowering the clock frequency down to 800 kHz, 6.4 MHz, and 40 kHz. The input supply voltage of this block is 1.8 V. In addition, the interface block controls the communication between the analog part and the digital part. The response is generated in a module in this block. In the module, 40 kHz clock signal is used to generate the response. To simplify Figure 1, this clock signal is not shown.

The comparator block compares the data such as the output of the hash function block and the data sent from the server. This block operates with 800 kHz clock signal and the input supply voltage is 1.8 V.

The finite state machine block controls the flow of OMHSO protocol. The flow consists of the following 8 steps: loading tag state and seed for PRNG from EEPROM to SRAM, loading key from EEPROM to SRAM, calculation of *α* and *β* with hash function, calculation of *Z*′ with hash function, comparing *Z* and *Z*′, calculation of new key with hash function, EEPROM block writing for new key, and EEPROM block writing for new seed. This block operates with 800 kHz clock signal and the input supply voltage is 1.8 V.

The volatile memory is a 128-byte SRAM (Static Random Access Memory) and is used to save temporal data such as the data received from the server, the secret data loaded from the nonvolatile memory, and the output of the hash function block. The data width of SRAM is 8 bits to achieve low-power consumption of the tag. This block operates with 800 kHz clock signal and the input supply voltage is 1.8 V.

The hash function block calculates the hash values of the input data. The hash functions implemented on this block are SPONGENT-160 and Keccak. These algorithms are switched and used. This block operates with 800 kHz clock signal and the input supply voltage is 1.8 V.

The interface of the hash function module is based on SRAM with 8-bit data width. Namely, the input data is cut into 8-bit blocks and the blocks are taken as an input to the hash function module one by one. Similarly, hash value is cut into 8-bit blocks and the blocks are returned from the hash function module one by one.

The hash function module has FF (Flip-Flops) called state to store the intermediate data. After the state is initialized with 0, the input blocks are XORed into a part of the state interleaved with the application of the permutation function of SPONGENT-160 or Keccak. At the end of every clock, the state is updated with the output of the permutation function. After absorbing all the input blocks, hash value is produced. A part of the state is returned as the hash block interleaved with the application of the permutation function. The state is updated continuously by the permutation function until desired bits of the hash value are returned.

The memory controller block communicates with SRAM and EEPROM for the data transfer. This block controls read/write operands, the address, and the data. This block operates with 800 kHz clock signal and the input supply voltage is 1.8 V.

Finally, the nonvolatile memory is a 1 Kbyte EEPROM (Electrically Erasable Programmable Read-Only Memory) and is used to save the secret keys and the seed needed for the random number generation. The input supply voltage is 3.3 V. This block operates with 6.4 MHz clock signal. Additional information about the blocks except the hash function block is described in [10].

## 4. Hardware Evaluation Using the Actual Tag

In this section, we show evaluation results of the tag in terms of cost, speed, power, and the communication distance. As two hash functions can be switched and used, this mechanism enables us to evaluate the performance of the tag depending on the security strength of the cryptographic hash functions.

### 4.1. Cost Evaluation

**Figure 2:**
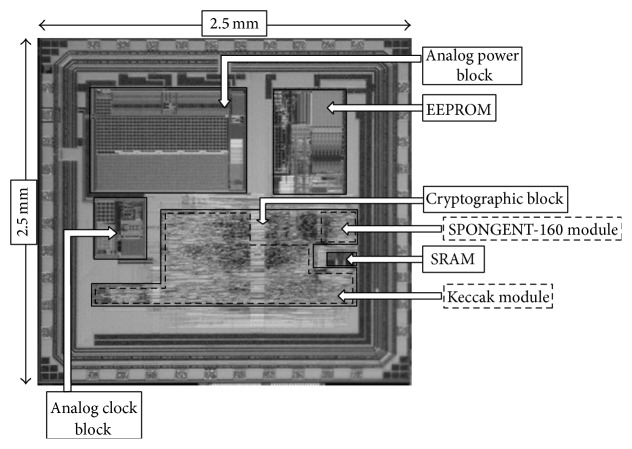
Micrograph of the RFID tag chip.

The whole RFID tag chip is fabricated in 180 *μ*m CMOS process.  Figure 2 shows the photograph of the manufactured RFID tag chip. As shown in Figure 2, the tag chip includes the analog power block, the analog clock block, the cryptographic block, 128-byte SRAM, and 1 Kbyte EEPROM. The RFID tag chip size is 2.5 mm × 2.5 mm. The reason for selecting a relatively large chip size is due to the number of I/O pins required for the performance evaluation.

Table 1 shows the area requirements of the cryptographic block in the RFID tag chip. The results are based on the synthesis result and shown in both area and gate equivalent (GE) manner. The gate equivalent is calculated on the basis of the area a 2-input NAND gate occupies, that is, 8.78 *μ*m^2^.

The area requirements of the interface module are due to the functionalities of the clock divider, the reader command decoder, and the signal formation of the tag response. The area requirements of the cryptographic block occupied with SPONGENT-160 are 2.9 kGE. The area requirements of the cryptographic block occupied with Keccak are 32.4 kGE.

Table 2 shows the area requirements of each block in the layout of the RFID tag chip. The analog power block takes the largest portion of the area. Since the analog power block has buffer capacitors, these capacitors take a large portion of the area. EEPROM also takes a large portion of the area. However, EEPROM that we used has much larger memory size than the required memory size needed to perform the authentication protocol.

### 4.2. Speed Evaluation

Table 3 shows the operation time for each step of OMHSO protocol using SPONGENT-160 or Keccak. When SPONGENT-160 is used, the tag chip generates the first response to the reader within 5 ms. The tag chip finishes all the operations within 20 ms. The hash calculations and the EEPROM block write operations take large part of the time.

Similarly, when Keccak is used, the tag chip generates the first response to the reader within 0.3 ms. The tag chip finishes all the operations within 10 ms. The EEPROM block write operations take large part of the time.

Next, we evaluate the time needed to run the whole process of OMHSO protocol including communication between the tag and a reader. The length of Tari is 25 *μ*s in the wireless communication between the tag and the reader. The average data rate for the reader-to-tag communication is 27 kbps. The average data rate for the tag-to-reader communication is 95 kbps. Since we set the data length as described in Section 3.2, the tag and the reader send 224-bit data to each other. Thus the predicted time needed to exchange the data is 10.7 ms in total.

Therefore, the time required to complete OMHSO protocol using SPONGENT-160 is less than 30 ms. Similarly, the time required to complete OMHSO protocol using Keccak is less than 20 ms.

The tag using Keccak finishes authentication process about 9 ms faster than the tag using SPONGENT-160. Since the time required for the processes without hash calculation is independent from the hash functions, the difference of the time results from the hash calculations.

While the number of clock cycles needed for the cryptographic process of Keccak is smaller than that of SPONGENT-160, the cryptographic process of Keccak is more complex than that of SPONGENT-160. As shown in Section 4.1, the area requirements of SPONGENT-160 are smaller than that of Keccak. Therefore, there is a trade-off between the area requirements and the time.

### 4.3. Power Evaluation

#### 4.3.1. Simulated Power Consumption after Layout

We show the average power consumption of the cryptographic block based on a postlayout simulation. We use Synopsys (Synopsys is a registered trademark of Synopsys Inc.) Design Compiler (Design Compiler is a product name of Synopsys Inc.) (Version C-2009.06-SP5) and TSMC (TSMC is a registered trademark of Taiwan Semiconductor Manufacturing Company Limited) 90 nm enhanced (CLN90G) process, Low-K dielectric 1.0 V SAGEX(tm) v3.0 standard cell library.

The baseband input signal is prepared in the simulation and is taken as the input to the cryptographic block. The postlayout netlist and the gates information are used to generate the signal toggle information. Based on these power consumption profiles, the average power consumption for each step of OMHSO protocol is evaluated.

Table 4 shows the average power consumption of the cryptographic block using SPONGENT-160. Similarly, Table 5 shows the average power consumption of the cryptographic block using Keccak. In Tables 4 and 5, step is the same as the one used in Table 3. In this simulation, the power consumption of SRAM and EEPROM is not included since the power consumption of these components cannot be evaluated correctly by the same method.

In Tables 4 and 5, the hash function module is active in Steps (3), (4), and (6). When the hash function module is active, the hash function module consumes about double or three times the power. The interface module, which includes the clock divider, constantly consumes about 150 *μ*W power. The interface module takes the largest part of the power consumption of the cryptographic block when SPONGENT-160 is used. On the other hand, when Keccak is used, the hash function module takes the largest part of the power consumption of the cryptographic block.

#### 4.3.2. Power Consumption of the Chip with RF-Based Power Supply

In this section, we show evaluation results of the tag in terms of power consumption of the digital processing block including SRAM and EEPROM. We deduce that it is very important to use a single tag chip for evaluation since the performance of the tag chips is individually different. Therefore, we use the evaluation board of the chip to switch and use the hash functions on a single tag chip.

**Figure 3:**
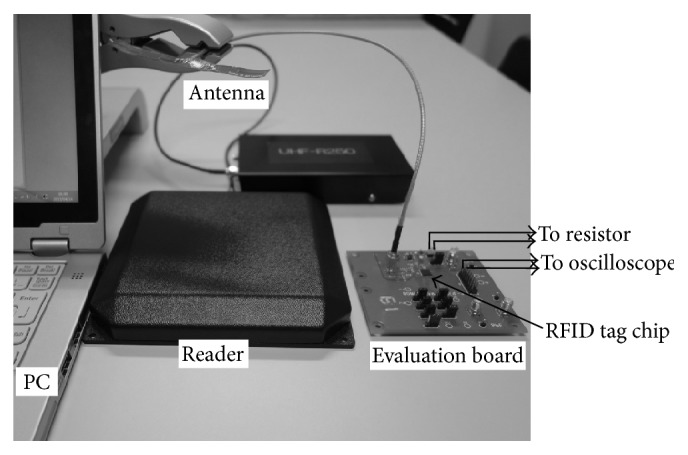
Power evaluation system of RFID tag chip.

Next, we describe the evaluation methodology.  Figure 3 shows the evaluation system of the RFID tag chip using the evaluation board. The board has a debug functionality. The debug functionality enables us to observe the voltage via the debug pins. The functionality is set by a dip switch on the board.

There are two methods to supply power to the evaluation board: an external stable power supply and RF-based power supply. Originally, a passive tag harvests the necessary power from the RF field. To evaluate the performance of the tag under actual operating conditions, we decided to supply power to the chip from the RF field. We connect the antenna that is connected to an SMA connector on the evaluation board via cable. We attach the cable to the height adjustment device so that the distance between the antenna and the reader is variable. The data communicated between the reader and the tag are controlled on PC. The signal level is set to 24 dBm (250 mW) for the output of the reader.

For power evaluation of the digital processing block including SRAM, we use an oscilloscope to measure the voltage drop in the 1.8 V power supply line. We add several resistors in the 1.8 V power supply line, which is connected to the digital processing block, to measure the voltage drop. Next, we calculate the electric current by dividing the voltage drop by the resistance. Then, we evaluate the power consumption of the digital processing block by multiplying the calculated electric current and the voltage after the resistor.

**Figure 4:**
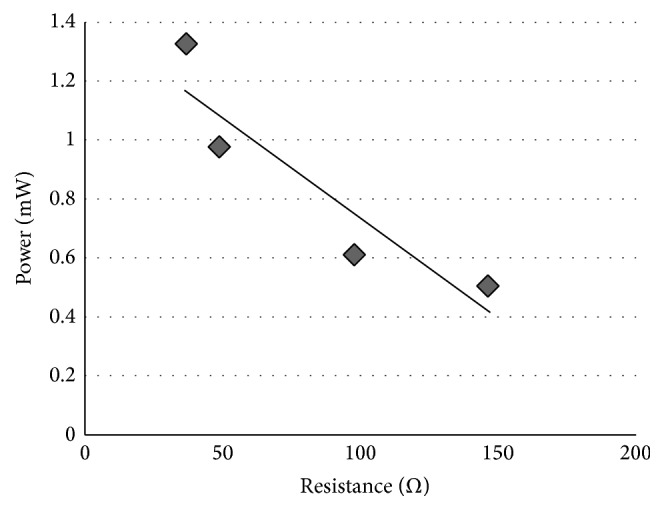
Peak power consumption of the digital processing block using SPONGENT-160.

**Figure 5:**
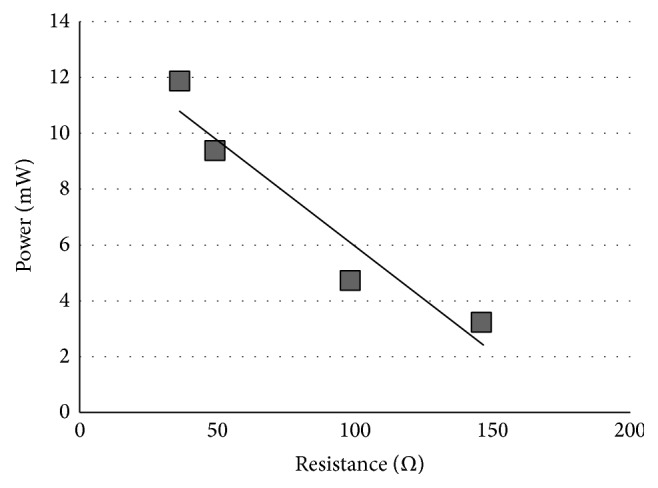
Peak power consumption of the digital processing block using Keccak.

In Figure 4, we plot the peak power consumption of the digital processing block using SPONGENT-160 versus the resistance. Similarly, Figure 5 shows the peak power consumption of the digital processing block using Keccak versus the resistance.

Now we evaluate the real peak power consumption of the digital processing block with these figures. From these figures, it seems that there is a linear relationship between the power consumption and the resistance. Usually the tag runs OMHSO protocol without the resistors. Thus, the actual power consumption of the block is the power that is measured under the condition that the added resistor will be 0 Ω. The actual values are equal to the intercepts of Figures 4 and 5. The intercepts of Figures 4 and 5 are 1.6 mW and 14.3 mW, respectively.

In the same way, we evaluate the power consumption of EEPROM when the authentication process is executed. In this case, we measure the voltage drop in the 3.3 V power supply line. The peak power consumption of EEPROM is 3.5 mW.

### 4.4. Communication Distance Evaluation

In this section, we show evaluation results of the maximum read distance between the tag and the reader. We first show our evaluation methodology. Similar to the power evaluation, we use the evaluation system shown in Figure 3. On this evaluation, we turn off the debug functionality and remove the resistor added in the power supply line since there is no need to measure the voltage drop.

We first set the distance between the reader and the antenna. Then the tag runs OMHSO protocol 50 times repeatedly and we count the number of successes. To stabilize the wireless communication between a tag and a reader, a commercial reader sends the command repeatedly depending on the application or communication environment of the tag. Thus, we decided to judge whether the communication is successful or unsuccessful by sending the command several times. The success probability is calculated by dividing the number of the successes by the number of the executions of the protocol.

**Figure 6:**
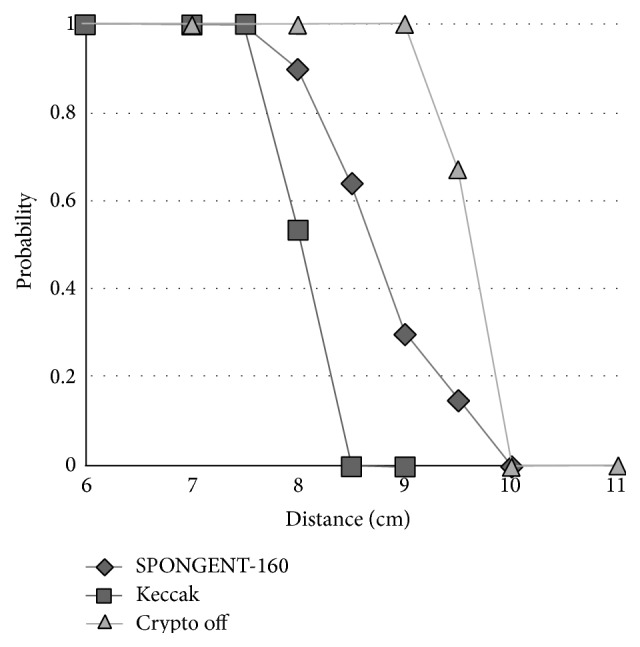
The communication distance between the tag and a reader.

We plot the communication distance and the success probability of the communication of each hash function in Figure 6. Figure 6 shows that the maximum read distance of the tag using SPONGENT-160 is 1.5 cm longer than that of the tag using Keccak.

However, it remains the possibility of error in the measured maximum read distance according to the electromagnetic wave conditions such as the reflected waves. To evaluate the error, we repeatedly performed the above-mentioned experiment. We focus on the distance where the success probability is 0 and it is close to 0. Our experimental results show that the number of the successes varies at the distance where the success probability is close to 0. On the other hand, the number of the successes is always 0 where the success probability is 0. These results show that the difference of the maximum read distance, which depends on the hash functions, is significant.

### 4.5. Discussion

As described in Section 4.3, the peak power consumption of EEPROM is larger than that of the digital processing block using SPONGENT-160. On the other hand, the peak power consumption of the digital processing block using Keccak is larger than that of EEPROM. Thus, when the lightweight hash function is used, the power consumption of EEPROM is a bottleneck. Similarly, when the standard hash function is used, the power consumption of the hash function is a bottleneck.

Recently, low-power memories such as FRAM (Ferroelectric Random Access Memory) are being developed [32]. In the future, when these memories will be used widely and implemented in tags, the power consumed by modules except the cryptographic module is predicted to be smaller. Thus, these memories will extend the maximum read distance when the lightweight cryptographic algorithm is used. On the other hand, when the standard hash function is used, the distance will not be longer since the most power consuming process is hash calculation.

## 5. Conclusion

In this work, we design, implement, and evaluate performance of a fully integrated passive UHF RFID tag that runs a mutual authentication protocol based on hash functions that preserve privacy. We design a single chip including the RF front end and the digital processing block. We choose lightweight hash function SPONGENT-160 supporting 80-bit security strength and standard hash function Keccak supporting 128-bit security strength. We implement all modules that are needed to run the whole authentication process including SRAM and EEPROM. Our evaluation results show that the maximum read distance is 10 cm when the lightweight hash function is used. The time required for the first response of the tag including a hash calculation is less than 5 ms and the authentication is completed within 30 ms. The area requirements of the cryptographic block are 10 kGE. The average power consumption of the cryptographic block is 260 *μ*W when the hash function is active. The peak power consumption of the tag is 3.5 mW that is consumed by EEPROM access. From our evaluation results, the RFID mutual authentication protocol based on a hash function is feasible for the passive UHF RFID tag.

When the standard hash function is used, the maximum read distance is 8.5 cm. The time required for the first response of the tag including a hash calculation is less than 0.3 ms and the authentication is completed within 20 ms. The area requirements of the cryptographic block are about 39.4 kGE. The average power consumption of the cryptographic block is 536 *μ*W when the hash function is active. The peak power consumption of the tag is 14.3 mW that is consumed by the hash calculation.

The maximum read distance of the tag with the lightweight hash function is longer than that of the tag with the standard hash function. As the most power consuming process of the tag using the lightweight hash function is EEPROM access and this power will be smaller when a low-power memory will be used, the maximum read distance will be longer when the memory will be used. On the other hand, as the most power consuming process of the tag using the standard hash function is the hash calculation and this power does not depend on the memory, the maximum read distance will not be longer when the memory will be used.

## Figures and Tables

**Algorithm 1:**
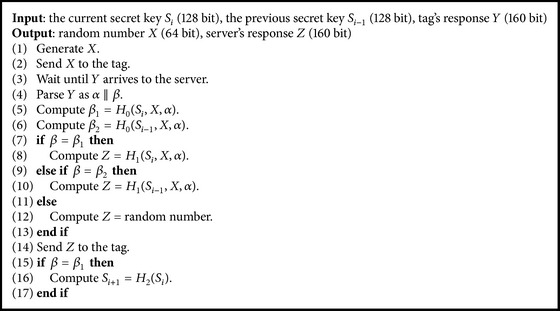
Authentication process of the server.

**Algorithm 2:**
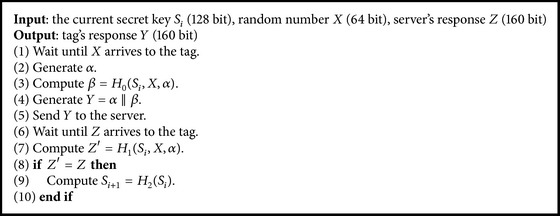
Authentication process of the tag.

**Table 1:** Area and GE (gate equivalent) of modules in the cryptographic block after synthesis.

Component	Area (μm^2^)	GE
Interface	50340	5734
SPONGENT-160 (Hash 1)	25809	2940
Keccak (Hash 2)	284641	32419
Others	11885	1353

Total	372675	42446

**Table 2:** Area of blocks on the RFID tag chip after layout.

Component	Area (μm^2^)
Analog power block	811915.6
Analog clock block	150955.7
Cryptographic block	372675
Volatile memory (SRAM)	39372.1
Nonvolatile memory (EEPROM)	398950

Total	1773868.4

**Table 3:** Time evaluation of OMHSO protocol using SPONGENT-160 or Keccak on the tag. The results using SPONGENT-160 are already mentioned in [10].

Step	Process	Time (ms)	Time (ms)	Difference of time (ms)
	Hash function	SPONGENT-160	Keccak	—

(1)	Load tag state and seed for PRNG from EEPROM to SRAM	0.02	0.02	0
(2)	Load key from EEPROM to SRAM	0.02	0.02	0
(3)	Calculate *α* and *β* with hash function	4.52	0.23	4.29

(4)	Calculate *Z*′ with hash function	2.94	0.14	2.8
(5)	Compare *Z* with *Z*′	0.06	0.06	0
(6)	Calculate new key with hash function	2.04	0.11	1.9
(7)	EEPROM block write for new key	4.33	4.33	0
(8)	EEPROM block write for new seed	4.33	4.33	0

	Total	18.26	9.24	9.02

**Table 4:** Simulated power consumption of the cryptographic block using SPONGENT-160 (*μ*W).

Step	(1)	(2)	(3)	(4)	(5)	(6)	(7)	(8)

Interface	157.0	155.0	154.0	154.0	153.0	153.0	153.0	153.0

SPONGENT-160	19.4	19.4	39.5	39.5	19.8	39.5	19.4	19.4

Keccak	1.1	1.1	1.1	1.1	1.2	1.1	1.1	1.1

Others	83.5	84.5	25.4	26.4	88.0	112.4	25.5	25.5

Total	**261.0 **	**260.0 **	**220.0 **	**221.0 **	**262.0 **	**220.0 **	**199.0 **	**199.0**

**Table 5:** Simulated power consumption of the cryptographic block using Keccak (*μ*W).

State	(1)	(2)	(3)	(4)	(5)	(6)	(7)	(8)

Interface	148.0	146.0	147.0	147.0	147.0	148.0	145.0	144.0

SPONGENT-160	0.1	0.1	0.1	0.1	0.1	0.1	0.1	0.1

Keccak	132.0	132.0	315.0	283.0	130.0	340.0	130.0	130.0

Others	76.9	77.9	48.9	80.9	80.9	47.9	18.9	19.9

Total	**357.0 **	**356.0 **	**511.0 **	**479.0 **	**358.0 **	**536.0 **	**294.0 **	**294.0**
